# Dermatomyositis does not adversely affect functional outcomes after total hip arthroplasty: a 1:2 matched case-control study

**DOI:** 10.1186/s42836-026-00400-y

**Published:** 2026-06-04

**Authors:** Hongjun Xu, Zhongyin Ji, Songlin Li, Yang Yu, Mingyang Ma, Guiguan Wang, Zhaojing Yin, Yiyang Du, Sen Liu, Wenwei Qian

**Affiliations:** 1 Department of Orthopedic Surgery, Peking Union Medical College Hospital, Peking Union Medical College and Chinese Academy of Medical Science, Beijing, 100730 China; 2 Department of Orthopedics, Qilu Hospital of Shandong University, Jinan, 250012, China

**Keywords:** Total hip arthroplasty, Osteonecrosis of femoral head, Dermatomyositis, Hip function, Respiratory complications, Pulmonary function

## Abstract

**Background:**

Patients with dermatomyositis (DM) are predisposed to osteonecrosis of the femoral head (ONFH), frequently requiring total hip arthroplasty (THA). However, limited evidence exists regarding postoperative outcomes in this population. This study aimed to evaluate functional outcomes and complication rates after THA in patients with DM compared to a matched cohort of patients without DM.

**Methods:**

A retrospective case-control study was conducted using our institution’s arthroplasty registry. We identified 18 patients (21 operations) with DM who underwent primary THA and matched them in a 1:2 ratio to 36 patients (42 operations) who underwent THA for hip osteoarthritis, alcohol-induced ONFH, or Crowe I-II developmental dysplasia of hips (DDH). Functional outcomes and health-related quality of life (QoL) were assessed, and postoperative complications were recorded.

**Results:**

At a mean follow-up of 4.87 years, both cohorts demonstrated comparable improvements in all functional and QoL measures (all postoperative comparisons, *p* > 0.05). The rate of complications, including dislocation, periprosthetic joint infection, and revision, was not significantly different between groups. However, respiratory complications occurred more frequently in the DM cohort (4/21 vs. 0/42, *p* = 0.010). Within the DM group, postoperative respiratory complications showed a preliminary association with higher preoperative D-dimer levels (*p* = 0.040).

**Conclusions:**

Patients with DM can achieve excellent functional outcomes after THA, comparable to those of patients without rheumatic autoimmune disease. Although most complication rates are similar, DM patients have an elevated risk of postoperative respiratory complications. Preoperative assessment of D-dimer levels and pulmonary function, along with proactive postoperative respiratory rehabilitation, may help reduce this risk in this vulnerable population.

## Background

Dermatomyositis (DM) is a rare systemic autoimmune inflammatory myopathy characterized by distinctive cutaneous manifestations and variable degrees of skeletal muscle inflammation [1, 2]. Management of DM often requires long-term administration of systemic glucocorticoids and immunosuppressive agents to control disease activity [2]. A well-established complication of prolonged corticosteroid therapy is osteonecrosis of the femoral head (ONFH), leading to severe hip pain, functional limitation, and a significant decline in quality of life [3, 4].

For patients with end-stage ONFH, total hip arthroplasty (THA) is the gold-standard surgical treatment, proven to be highly effective in alleviating pain, restoring mobility, and improving quality of life [5]. While the benefits of THA in the general population are well-established, its clinical outcomes in patients with DM remain unknown. Concerns exist that the underlying systemic inflammation, chronic muscle weakness, and long-term immunosuppressive therapy could predispose these patients to inferior postoperative functional recovery and a higher incidence of perioperative complications, such as periprosthetic joint infection (PJI) and impaired wound healing [6]. A previous study reported that patients with DM or polymyositis had increased rates of postoperative pneumonia and pulmonary embolism after THA [6]. However, that study lacked detailed postoperative functional outcomes or a matched control group for comparison.

**Fig. 1:**
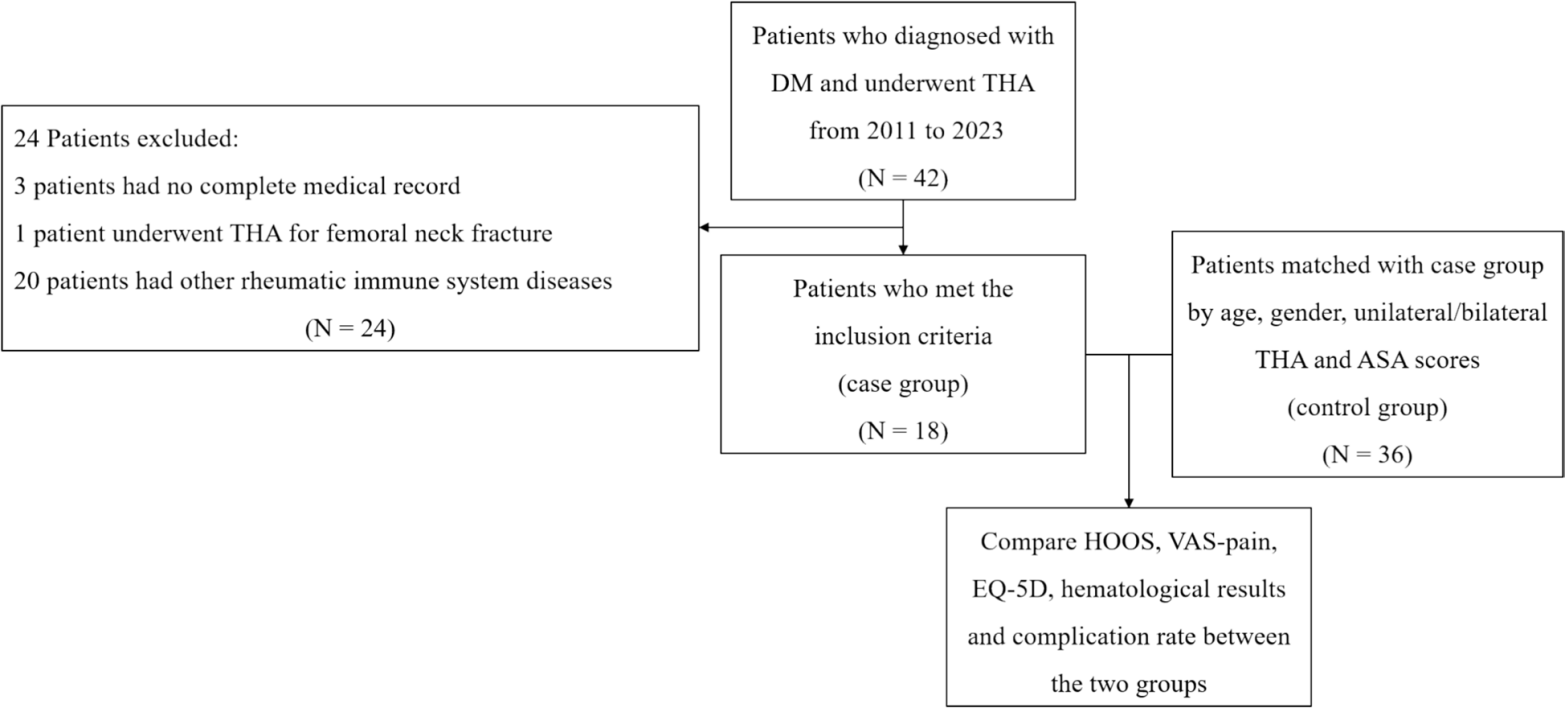
Flowchart of patient selection process

Understanding the specific risks and expected functional recovery after THA is essential for optimizing perioperative management for patients with DM. Therefore, this study was designed to address this knowledge gap. We aimed to compare outcomes of THA in patients with DM to those in a matched control group without DM to evaluate: (1) postoperative hip function and pain relief; (2) the incidence of perioperative complications; and (3) the risk factors associated with postoperative respiratory complications in the DM group (Fig. 1).

## Methods

### Study design and participants

This retrospective case-control study was conducted by reviewing our single institution’s maintained arthroplasty registry. We identified all patients with a confirmed diagnosis of DM and ONFH who underwent primary THA between January 2011 and December 2022. DM is a major subgroup of the broader category of idiopathic inflammatory myopathies (IIMs). In this study, all included patients were diagnosed with IIM and further specifically subclassified as DM according to the 1975 Bohan and Peter criteria or the 2017 EULAR/ACR classification criteria [7, 8]. Specifically, the diagnosis of DM required the definitive presence of characteristic cutaneous manifestations (such as Gottron’s papules, Gottron’s sign, or heliotrope rash) combined with varying degrees of proximal muscle weakness, elevated skeletal muscle enzymes, compatible electromyographic findings, or muscle-biopsy evidence. All diagnoses were confirmed and documented by senior rheumatologists at our institution. Patients were excluded if they had incomplete medical records, concomitant systemic rheumatic diseases, or underwent THA for hip fractures. This process yielded a case group of 18 patients. Among them, 13 patients underwent unilateral THA, two patients underwent simultaneous bilateral THAs (representing 2 operations), and three patients underwent staged bilateral THAs (representing 6 operations). For the patients who underwent staged bilateral THAs, the median and IQR of the interval between the two operations were 5 (4–12) months. Therefore, a total of 21 operations were included in the case group for the analysis of perioperative complications.

To mitigate selection bias and control confounding factors, a control group was established by matching patients without systemic rheumatic diseases from our registry who underwent primary THA for hip osteoarthritis, alcohol-induced ONFH, or Crowe I-II developmental dysplasia of the hip (DDH) during the same period. A 1:2 individual matching approach was employed to establish the control cohort. Each patient with DM was manually matched to two specific control patients based on the following criteria: age (± 1 year), gender, American Society of Anesthesiologists (ASA) physical status classification score (± 1 score), laterality of surgery (unilateral, simultaneous bilateral, or staged bilateral), and duration of follow-up (± 2 years). This matching process yielded a control cohort of 36 patients. Among them, 26 patients underwent unilateral THA, four patients underwent simultaneous bilateral THAs (representing 4 operations), and six patients underwent staged bilateral THAs (representing 12 operations). The median and IQR of the interval for staged surgeries in the control group were 7 (6–8) months. The total number of operations analyzed in the control group was 42. The study protocol was approved by our Institutional Review Board.

### Surgical procedure and perioperative management

All THA procedures were performed by a group of surgeons with high surgical volume using a standardized technique. A posterolateral approach was used in all cases, and cementless components were implanted. Perioperative management protocols were consistent for both groups. These protocols included multimodal pain management, standardized antibiotic prophylaxis, and chemical thromboprophylaxis consisting of low-molecular-weight heparin from postoperative day 1 (POD 1) to POD 3, followed by aspirin from POD 4 to POD 35. All patients began a standardized physical therapy regimen on postoperative day 1. Patients were followed up routinely at 6 weeks, 6 months, 1 year, and annually thereafter.

### Data collection

Demographic data, including age, gender, body mass index (BMI), and ASA score, were extracted from our electronic medical record system. For the case group, we also collected information on the age at diagnosis of DM, duration of DM, presence of interstitial lung disease (ILD), and relevant laboratory parameters. Detailed pharmacological histories were also retrospectively extracted from the medical records. This included the total continuous duration of corticosteroid therapy prior to surgery, the current preoperative corticosteroid dosage (which was standardized to a daily prednisone equivalent dose in mg/day), and the specific treatment intensity of disease-modifying antirheumatic drugs (DMARDs) (categorized as either monotherapy or combination therapy involving ≥ 2 agents). Operative time and length of postoperative hospital stay were recorded for all patients.

Functional outcomes were assessed using patient-reported outcome measures (PROMs) collected preoperatively and at the latest follow-up. The primary functional measurement was the Hip disability and Osteoarthritis Outcome Score (HOOS), a patient-reported outcome measure [9]. The European Quality of Life 5-Dimension scale (EQ-5D) and the European Quality of Life-visual analog scale (EQ-VAS) were used to assess the health-related quality of life [10]. Pain intensity was also evaluated using a Visual Analog Scale (VAS) in response to the question: “How intense is your pain right now?” with a scale ranging from 0 (no pain) to 10 (the most intense pain).

Postoperative complications were identified and recorded through the medical record system. Complications included incision-related complications (superficial infection, delayed wound healing, hematoma), vascular or nerve injury, high fever (body temperature > 39 °C), prosthetic hip dislocation, PJI, pulmonary or urinary tract infection within 30 days after THA, postoperative hypoxemia (arterial PaO₂ < 80 mmHg) in the absence of pulmonary infection or pulmonary embolism (PE), and deep vein thrombosis (DVT) or PE within 30 days after surgery diagnosed by color Doppler ultrasound of the lower-extremity vessels and computed tomography angiography of the pulmonary arteries. Any subsequent reoperations, revisions, and 90-day hospital readmissions were also documented. Postoperative respiratory complications were defined as the occurrence of any pulmonary infection, pulmonary embolism, or hypoxemia after surgery.

Hematological parameters, including preoperative, POD 1, and POD 3 hemoglobin (Hb) and hematocrit (Hct) levels, were collected. Total estimated blood loss was calculated using the Gross formula [11]. Preoperative and postoperative serum albumin levels were also collected.

### Statistical analysis

Statistical analyses were performed using SPSS version 25.0 (IBM Corp., Armonk, NY, USA). Although an individual matching approach was used for cohort selection to ensure optimal baseline comparability, the case and control groups were analyzed as independent cohorts using unpaired statistical tests. Continuous variables with normal distribution were described as mean ± standard deviation (SD), and the unpaired t-test was used to assess differences between the case group and the control group in these variables. Continuous variables with non-normal distribution were presented as median and interquartile range (IQR), and the Mann–Whitney test was used. Categorical variables were compared using the chi-squared test or Fisher’s exact test. The *p*-value < 0.05 was considered statistically significant for all analyses.

Outcomes were analyzed at either the patient level or the operation level. Patient-level analysis (*n* = 18 for the DM group; *n* = 36 for the control group) was applied to the outcomes, including gender, age, BMI, and ASA score. Operation-level analysis (*n* = 21 for the DM group; *n* = 42 for the control group) was utilized for the outcomes, including operation time, length of postoperative hospital stays, follow-up time, HOOS, VAS pain score, EQ-5D, EQ-VAS, hematological index, and perioperative complications. Simultaneous bilateral THAs were counted as a single operation for analysis. While staged bilateral THAs were treated as two distinct operations. Each stage had its own independent 30-day postoperative time window for monitoring complications.

## Results

### Demographic and operative characteristics

Our study included 18 patients (21 operations) in the case group and 36 patients (42 operations) in the control group. These two groups were well-matched, with no significant differences in age, gender distribution, BMI, or ASA score (*p* > 0.05). The mean follow-up duration was 4.48 ± 1.5 years for the case group and 5.07 ± 1.73 years for the control group (*p* = 0.185). There were no significant differences in operative time or length of postoperative hospital stay between the groups (both *p* > 0.05). Detailed demographic and operative data are presented in Table 1.

**Table 1:** Demographic characteristics

	**Total**	**Case group**	**Control group**	***p*-value**
Patients (*n*)	54	18	36	-
Female sex (*n* [%])		12 (66.7)	24 (66.7)	1.000
Age (years)		42.19 ± 16.13	42.12 ± 15.84	0.987
Bilateral surgery (*n* [%])		2 (11.1)	4 (11.1)	1.000
Staged surgery (*n* [%])		3 (16.67)	6 (16.67)	1.000
BMI (kg/m^2^)		24.05 ± 3.43	23.88 ± 3.65	0.862
ASA score		1.86 ± 0.48	1.81 ± 0.40	0.677
Operation time (min)		100 ± 44.86	98.52 ± 36.84	0.890
Length of postoperative hospital stay (days)		6.86 ± 4.42	5.55 ± 1.64	0.202
Follow-up time (years)		4.48 ± 1.50	5.07 ± 1.73	0.185

*Abbreviations: BMI* body mass index, *ASA* American Society of Anesthesiologists

### Hip functional and pain outcomes

Both groups demonstrated marked improvements in all functional and pain scores from preoperative evaluation to the final follow-up. Preoperatively, there were no significant differences in any HOOS subscale, VAS pain score, or EQ-5D subscale (*p* > 0.05 for all). Postoperatively, there were no statistically significant differences between the case group and control group in HOOS-Pain, HOOS-Symptoms, HOOS-ADL, HOOS-SR, HOOS-QoL, or total HOOS scores. Similarly, postoperative EQ-5D scores and their subscales, as well as EQ-VAS values, were comparable between the groups. Postoperative VAS pain scores were low and not significantly different between cohorts. Detailed comparisons are presented in Tables 2 and 3.

**Table 2:** Hip function outcomes

	**Case group**	**Control group**	***p*-value**
**Preoperative**			
HOOS-symptom	50 ± 14.72	51.72 ± 11.68	0.687
HOOS-pain	36.72 ± 6.44	39.61 ± 8.21	0.190
HOOS-ADL	31.52 ± 11.89	35 ± 7.45	0.295
HOOS-SR	12.89 ± 9.26	8.55 ± 11.72	0.101
HOOS-QoL	21.88 ± 10.7	25.98 ± 8.85	0.310
HOOS-total	32.3 ± 8.68	35.7 ± 7.03	0.186
VAS pain score	7.31 ± 0.79	6.88 ± 0.79	0.104
**Postoperative**			
HOOS-symptom	87.5 ± 9.31	85.47 ± 7.76	0.271
HOOS-pain	93.28 ± 6.17	93.91 ± 5.35	0.867
HOOS-ADL	88.85 ± 7.02	87.2 ± 5.86	0.425
HOOS-SR	80.47 ± 10.67	80.27 ± 7.95	0.973
HOOS-QoL	86.33 ± 8.3	85.14 ± 7.55	0.685
HOOS-total	88.7 ± 6.58	87.76 ± 5.23	0.686
VAS pain score	1.13 ± 0.89	1.31 ± 0.97	0.569

*Abbreviations: HOOS* Hip disability and Osteoarthritis Outcome Score, *ADL* Activities of Daily Living, *SR* Sport and Recreation Function, *QoL* Quality of Life, *VAS* visual analog scale

**Table 3:** Health-related quality of life assessment outcomes

	**Case group**	**Control group**	***p*-value**
**Preoperative**			
EQ-mobility	3.38 ± 0.72	3.25 ± 0.80	0.602
EQ-self care	2.38 ± 0.66	2.56 ± 0.96	0.432
EQ-usual activity	2.94 ± 0.85	3.06 ± 0.72	0.595
EQ-pain	3.63 ± 0.89	3.72 ± 0.96	0.745
EQ-anxiety	2.94 ± 1.12	2.78 ± 1.01	0.628
EQ-scores	15.44 ± 2.43	15.19 ± 2.36	0.732
EQ-VAS	59.38 ± 12.5	64.28 ± 16.83	0.308
**Postoperative**			
EQ-mobility	1.69 ± 0.60	1.72 ± 0.52	0.854
EQ-self care	1.38 ± 0.50	1.16 ± 0.37	0.093
EQ-usual activity	1.19 ± 0.40	1.28 ± 0.46	0.49
EQ-pain	1.38 ± 0.50	1.41 ± 0.49	0.839
EQ-anxiety	1.13 ± 0.34	1.25 ± 0.44	0.325
EQ-scores	6.75 ± 1.53	6.81 ± 1.18	0.876
EQ-VAS	80.81 ± 7.16	79.50 ± 6.83	0.540
Patient satisfaction	95.63 ± 4.32	96.16 ± 4.15	0.682

### Hematological outcomes

Hematological results of the two groups are summarized in Table 4. No significant differences were observed between the case and control groups in preoperative hemoglobin (135.81 ± 15.02 g/L vs. 139.38 ± 13.47 g/L, *p* = 0.430) or postoperative hemoglobin levels on POD 1 and POD 3 (*p* = 0.371 and *p* = 0.414, respectively). Similarly, hematocrit values measured preoperatively and on POD 1 and POD 3 were comparable between groups (all *p* > 0.05). Total perioperative blood loss did not differ significantly between the case and control groups (1119.35 ± 440.21 mL vs. 1036.4 ± 311.38 mL, *p* = 0.507). Although patients in the case group showed slightly lower preoperative and postoperative serum albumin levels (42.44 ± 2.22 g/L vs. 43.84 ± 3.16 g/L and 31.38 ± 3.48 g/L vs. 32.66 ± 3.64 g/L, respectively), these differences were not statistically significant (*p* = 0.082 and *p* = 0.245, respectively).

**Table 4:** Hematological index

	**Case group**	**Control group**	***p*-value**
Preop. Hb (g/L)	135.81 ± 15.02	139.38 ± 13.47	0.430
Hb POD1 (g/L)	113.44 ± 15.45	117.81 ± 16.33	0.371
Hb POD3 (g/L)	103.25 ± 17.79	107.47 ± 13.85	0.414
Preop. Hct (%)	39.88 ± 4.56	41.07 ± 3.56	0.367
HCT POD1 (%)	34.13 ± 4.76	34.66 ± 4.23	0.707
HCT POD3 (%)	30.69 ± 4.90	31.8 ± 4.01	0.439
Total blood loss (mL)	1119.35 ± 440.21	1036.4 ± 311.38	0.507
Preop. Albumin (g/L)	42.44 ± 2.22	43.84 ± 3.163	0.082
Postop. albumin (g/L)	31.38 ± 3.48	32.66 ± 3.64	0.245

Abbreviations: Hb, hemoglobin; POD, postoperative day; HCT, hematocrit

### Complications

Postoperative complications are summarized in Table 5. The overall incidence of complications was low in both groups. No incision complications, dislocations, periprosthetic joint infections, revision surgery, reoperations, or readmissions within 90 days were observed in either group. One vascular and nerve injury and one urinary tract infection occurred in the control group, while none were observed in the case group (*p* = 0.999 for both).

**Table 5:** Perioperative complications

	**Case group (n/N)**	**Control group (n/N)**	***p*-value**
Incision complication (n)	0/21	0/42	1.000
Vascular and nerve injury (n)	0/21	1/42	0.999
High fever (n)	1/21	0/42	0.333
Dislocation (n)	0/21	0/42	1.000
Periprosthetic joint infection (n)	0/21	0/42	1.000
Pulmonary infection with 30 days (n)	2/21	0/42	0.108
Urinary tract infection with 30 days (n)	0/21	1/42	0.999
Deep vein thrombosis with 30 days (n)^†^	1/21	0/42	0.333
Pulmonary embolism with 30 days (n)^†^	1/21	0/42	0.333
Postoperative hypoxemia (n)	1/21	0/42	0.333
Respiratory complications (n)	4/21	0/42	0.010*
Revision surgery (n)	0/21	0/42	1.000
Transfusion (n)	3/21	3/42	0.391
Reoperation (n)	0/21	0/42	1.000
Readmission with 90 days (n)	0/21	0/42	1.000

Values presented in a format of “n/N”, where n represents the number of events, and N represents the total number of operations

^†^The one case of deep vein thrombosis and the one case of pulmonary embolism recorded in the case group occurred concurrently in the same patient

^*^It indicates statistical significance (*p* < 0.05)

Within the DM group, one patient developed a high fever, one experienced postoperative hypoxemia, one single patient suffered from concurrent DVT and PE, and two developed pulmonary infections within 30 days postoperatively, though these differences were not statistically significant compared with the control group (all *p* > 0.05). However, the incidence of total respiratory complications was higher in the case group (4/21 vs. 0/42, *p* = 0.010). No significant intergroup differences were noted in the rates of transfusion (all *p* > 0.05).

### Risk of postoperative respiratory complications

Univariate analysis of potential risk factors for postoperative respiratory complications among the 21 operations in DM patients is presented in Table 6. Among DM patients, 4 out of 21 operations developed postoperative respiratory complications. There were no significant differences between patients with and without respiratory complications in terms of age, gender, BMI, ASA score, operation time, or preoperative pain and PROMs (VAS-pain, HOOS, EQ-5D, EQ-VAS; all *p* > 0.05).

**Table 6:** Univariate analysis for postoperative respiratory complications among the 21 operations in DM patients

	**With respiratory complications**	**Without respiratory complications**	***p*-value**
Operations (n)	4	17	
Female sex (n)	2/4	12/17	0.574
Age (years)	55(30–63)	45(26–53)	0.462
Bilateral surgery (n)	0/4	2/17	0.999
BMI (kg/m^2^)	22.38(19.91–26.07)	23.83(20.69–27.37)	0.410
ASA score	2(2–3)	2(1–2)	0.203
Operation time (min)	85(70–110)	95(75–105)	0.698
Preop. VAS pain score	7(6–8)	7(7–8)	0.965
Preop. HOOS	31.24(24.84–42.34)	36.25(27.50–37.18)	0.698
Preop. EQ-scores	16.5(14.5–18.5)	14(13–16)	0.099
Preop. EQ-VAS	64(43.25–69.75)	64(48–72)	0.763
Duration of DM (years)	6(4–12)	4(3–6)	0.120
Age at diagnosis (years)	48(24–50)	42(21–48)	0.462
Preop. FEV1/FVC (%)	78(65.8–79)	82(78.5–83.5)	0.635
Preop. FEV1 (L)	1.81(1.46–1.96)	2.13(1.83–2.73)	0.065
Preop. FVC (L)	2.17(1.73–2.39)	2.51(2.24–3.37)	0.052
**Glucocorticoids details**			
Glucocorticoid (n)	4/4	17/17	1.000
Prednisone equivalent dose (mg/day)	5	5(5–6.25)	0.763
Corticosteroid duration (months)	48(36–66)	72(51–130)	0.081
**DMARDs regimen details**			
DMARDs (n)	3/4	15/17	0.489
Monotherapy (1 type of DMARD) (n)	2/4	13/17	0.544
Combination therapy (≥ 2 types) (n)	1/4	2/17	0.489
With interstitial lung disease (n)	3/4	8/17	0.603
WBC counts (× 10^9^/L)	7.82(5.08–8.11)	6.44(5.36–8.28)	0.763
PLT counts (× 10^9^/L)	213(192–239)	255(220–291)	0.120
LY counts (× 10^9^/L)	2.28(1.32–2.87)	1.23(0.98–1.69)	0.203
D-dimer (mg/L FEU)	0.72(0.49–0.98)	0.40(0.25–0.63)	0.040*
ESR	23(9–38)	16(9–27)	0.574
CRP (mg/L)	0.9(0.6–3.2)	4.5(1.9–6.2)	0.099
CK (U/L)	64(37–140)	39(30–62)	0.203
ALT (U/L)	16(13–20)	17(11–27)	0.829
AST (U/L)	22(18–29)	23(17–27)	0.999
LDH (U/L)	190(185–199)	196(179–226)	0.763
Anti-Jo-1 (n)	0/4	6/17	0.281
Anti-MDA5 + (n)	2/4	9/17	0.999
Anti-Ro-52 (n)	3/4	7/17	0.311

The subgroups “With” (*n* = 4) and “Without” (*n* = 17) respiratory complications represent the total 21 operations performed in the DM cohort

^*^It indicates statistical significance (*p* < 0.05)

*Abbreviations: FEV1* Forced Expiratory Volume in the first second, *FVC* forced vital capacity, *DMARDs*, disease-modifying antirheumatic drugs, *WBC*, white blood cell, *PLT* platelet, *LY* lymphocyte, *ESR* Erythrocyte Sedimentation Rate, *CRP* C-reactive Protein, *CK* Creatine Kinase, *ALT* Alanine Aminotransferase, *AST*, Aspartate Aminotransferase, *LDH* Lactate Dehydrogenase

The duration of DM and age at diagnosis of DM were comparable between groups (*p* = 0.120 and *p* = 0.462, respectively). Preoperative pulmonary function parameters, including forced expiratory volume in the first second (FEV1), forced vital capacity (FVC), and FEV1/FVC ratio, tended to be lower in patients who developed respiratory complications, and the differences in FEV1 and FVC approached but did not reach statistical significance (*p* = 0.065 and 0.052, respectively).

No significant differences were observed in the categorical use of glucocorticoids or DMARDs or the presence of ILD between the two groups. Furthermore, detailed pharmacological analysis revealed no statistically significant differences in the median preoperative prednisone equivalent dose (*p* = 0.763), the duration of corticosteroid therapy (*p* = 0.081), or the proportion of patients receiving combination DMARD therapy (*p* = 0.489). Similarly, preoperative laboratory parameters—including white blood cell (WBC) counts, platelet (PLT) counts, lymphocyte (LY) counts, erythrocyte sedimentation rate (ESR), C-reactive protein (CRP), liver enzymes, Lactate Dehydrogenase (LDH), and creatine kinase (CK) levels—did not differ significantly (all *p* > 0.05). The frequencies of myositis-specific antibodies (anti–Jo-1, anti–MDA5, and anti–Ro-52) were comparable between groups. However, preoperative D-dimer levels among DM patients suffering from postoperative respiratory complications were significantly higher than those of DM patients without respiratory complications (*p* = 0.040).

## Discussion

As the management of systemic autoimmune diseases improves, more patients are living longer lives, leading to an increased demand for orthopedic procedures like THA to address joint destruction [12]. This study is, to our knowledge, the first matched case-control study to specifically assess the functional outcomes and complications of THA in patients with DM. Our principal finding is that patients with DM achieve excellent and comparable functional improvements to patients with hip osteoarthritis, Crowe I-II DDH, or alcohol-induced ONFH without an increased risk of major surgical complications such as dislocation, infection, or revision. However, they are at a significantly higher risk of developing respiratory complications.

In our study, the substantial improvements in HOOS, EQ-5D, and VAS-pain scores observed in the case group are encouraging. Our study did not support the concern that chronic myopathy and potential muscle atrophy inherent to DM might compromise functional recovery. Our results provide reassurance to both surgeons and patients that THA is a highly beneficial procedure for end-stage hip disease in the context of DM. These results aligned with a meta-analysis conducted by Lu et al., which indicated that patients with systemic lupus erythematosus (SLE) showed similar improvements in hip function postoperatively compared to non-SLE patients [13]. A propensity-matched study conducted by Clark et al. compared the postoperative modified Harris Hip Scores (mHHS) between patients with hip osteoarthritis and those with rheumatoid arthritis who underwent THA [14]. Similarly, no significant difference was observed between the cohorts.

A key concern for surgery in patients with autoimmune diseases is the risk of complications due to immunosuppressive therapy [14–18]. Hirpara et al. investigated complications after THA between patients with or without antiphospholipid syndrome (APS). They found that patients with APS had a higher risk of postoperative pneumonia, urinary tract infection, anemia, DVT, and PE [16]. Another study, conducted by Ratnasamy et al., found that patients with Behcet syndrome undergoing THA experienced markedly higher postoperative adverse events, including PE, pneumonia, and urinary tract infection [17]. In a meta-analysis, Huang et al. found that SLE patients following THA were at an increased risk of DVT, wound infection, dislocation, periprosthetic fracture, revision, periprosthetic joint infection, in comparison with non-SLE patients following THA. While in our study, we found no significant increase in the rates of PJI, dislocation, revision, or other major surgical complications in the DM group with a mean follow-up of 4.48 years in comparison with the control group. This may be attributed to differences in disease mechanisms and therapeutic exposure between SLE and DM. SLE is characterized by immune complex-mediated systemic inflammation often involving joints, kidneys, connective tissues, and skin [19, 20]. Combined with prolonged high-dose corticosteroid and immunosuppressive therapy, SLE may result in osteoporosis and capsular laxity, thereby increasing the risk of periprosthetic fracture, prosthesis loosening, PJI, and dislocation. In contrast, the effect of DM is mainly limited to muscles and microvasculature, exerting relatively milder systemic effects on bone and soft tissue. These findings suggest that, with appropriate perioperative management, the risks associated with DM and its treatment can be effectively mitigated, allowing for outcomes comparable to the general arthroplasty population.

However, the most significant difference identified between the groups was the incidence of total respiratory complications (4/21 vs. 0/42, *p* = 0.010), a composite complication including pulmonary infection, postoperative hypoxemia, and PE. This observation was consistent with a previous study, which reported increased rates of postoperative pneumonia and PE in a combined cohort of DM and polymyositis patients following THA [6]. Our study substantiated this finding within a matched-cohort comparison, highlighting that this risk persists even when patients are well-matched for age, gender, and ASA status. In a further univariate analysis of risk factors within the DM cohort, we found no significant association between respiratory complications and DM duration, age at diagnosis, specific myositis-specific antibodies, with or without ILD, or the preoperative use of glucocorticoids or DMARDs. Although our univariate analysis did not demonstrate a statistically significant dose-response relationship between the exact preoperative corticosteroid dosage or DMARD regimens and postoperative respiratory complications, the intensity of immunosuppressive therapy remains a critical clinical concern. As highlighted by Zhu et al., patients with the anti-MDA5 antibody-positive exhibit a particularly high prevalence of ONFH [21]. This is largely attributed to the aggressive nature of this phenotype, which frequently necessitates early, high-dose corticosteroids combined with potent DMARDs to manage life-threatening complications like rapidly progressive ILD. While such intense regimens are essential for controlling primary systemic disease activity, they drastically accelerate the onset of secondary ONFH and inevitably leave the patient in a profoundly immunosuppressed state. Notably, we found that preoperative D-dimer levels were significantly higher in DM patients who developed respiratory complications (*p* = 0.040). These findings indicate that patients with dermatomyositis are prone to a hypercoagulable state, thereby increasing the risk of DVT and PE [22]. Moreover, elevated D-dimer levels may also reflect underlying systemic inflammation [23]. Besides, we did observe a strong trend toward lower preoperative pulmonary function, specifically in FEV1 (*p* = 0.065) and FVC (*p* = 0.052), among patients who developed respiratory complications. Although this did not reach statistical significance, likely owing to the small number of events (*n* = 4), it points to a clinically important hypothesis: preoperative pulmonary compromise may be the most critical factor predisposing DM patients to postoperative respiratory complications. This is pathophysiologically plausible, as DM is associated with both ILD and respiratory muscle weakness [24, 25]. Interestingly, in our results, DM patients who developed respiratory complications did not show higher rates of ILD. This unexpected finding may be attributed to the relatively high prevalence of ILD among Asian patients with DM, as well as the limited sample size in our subgroup analysis [26]. Based on our findings, we propose a comprehensive perioperative management protocol to minimize complications in patients with DM. First, preoperative assessment should be rigorous. Beyond routine laboratory tests, we recommend pulmonary function tests, D-dimer quantification, and arterial blood gas analysis, regardless of whether the patient has a diagnosis of ILD. Second, for patients with identified risks (such as low FEV1 or elevated D-dimer), enhanced perioperative management is warranted. This should include proactive aerosol inhalation therapy with budesonide, ipratropium bromide, and N-acetylcysteine, and respiratory muscle training both before and after surgery to optimize lung condition. Third, postoperative care should focus on thrombosis prevention and infection control. We strongly advocate for active ankle pump exercises and early ambulation to counteract the hypercoagulable state. Furthermore, clinicians should maintain vigilant monitoring of body temperature, physical signs of the lungs, and inflammatory markers to distinguish early postoperative complications.

This study has several strengths. The matched-cohort design minimizes the influence of key demographic confounders, strengthening the validity of our comparisons. We utilized validated, patient-reported outcome measures to provide a comprehensive assessment of function and quality of life. Furthermore, the detailed analysis of complications and the investigation into respiratory complications risk factors provide novel and clinically relevant insights.

Nevertheless, we acknowledge several limitations. First, the retrospective nature of the study introduces the possibility of information bias, although data were collected from a prospectively maintained registry. Second, the sample size is relatively small, which is a common challenge when studying rare diseases like DM. This may have limited our power to detect differences in rare complications and further investigate the risk factors of complications. Specifically, the risk factor analysis presented in Table 6 is purely exploratory due to the extremely small number of events (*n* = 4), and the* p* = 0.040 for D-dimer must be interpreted with extreme caution. Third, this is a single-center study, and our findings may not be generalizable to all practice settings. Fourth, we could not control for all potential pharmacological confounders. While we successfully analyzed the preoperative corticosteroid dosage and DMARDs regimens, the precise cumulative historical duration of specific DMARDs exposure was inconsistently documented in our retrospective orthopedic registry. Fifth, the mean follow-up period of 4.48 years represents short-to-mid-term outcomes. Given that many patients with DM receive long-term immunosuppressive or glucocorticoid therapy, they may remain at elevated risk for late complications, such as late PJI or prosthesis loosening secondary to poor bone quality. Therefore, long-term survivorship in this population remains to be confirmed by future studies with extended follow-up durations. Further multicenter prospective studies with larger sample sizes and longer follow-up are needed to confirm our findings and further refine the optimal perioperative management for this unique patient population.

## Conclusions

In conclusion, this matched-cohort study demonstrated that patients with dermatomyositis who underwent total hip arthroplasty can achieve excellent pain relief and functional improvement, which are comparable to those of patients without rheumatic autoimmune diseases. The rates of major surgical complications, including PJI and revision, were not significantly increased in the DM cohort. However, these patients were at a significantly higher risk of postoperative respiratory complications, a risk that may be associated with preoperative D-dimer levels and potentially with preoperative FEV1 and FVC. These findings support that THA is a safe and effective treatment for end-stage hip disease in patients with DM, requiring strict perioperative attention to respiratory complications.

## Data Availability

The datasets generated and/or analysed during the current study are stored on the secure local server of Peking Union Medical College Hospital. The data are available from the corresponding author on reasonable request for non-commercial research purposes.

## Abbreviations

DM: Dermatomyositis
ONFH: Osteonecrosis of the femoral head
THA: Total hip arthroplasty
PJI: Periprosthetic joint infection
DDH: Developmental dysplasia of the hip
ASA: American Society of Anesthesiologists
POD: Postoperative day
BMI: Body mass index
DMARDs: Disease-modifying antirheumatic drugs
ILD: Interstitial lung disease
PROMs: Patient-reported outcome measures
HOOS: Hip disability and Osteoarthritis Outcome Score
EQ-5D: European Quality of Life 5-Dimension scale
QoL: Quality of life
VAS: Visual analog scale
PE: Pulmonary embolism
DVT: Deep vein thrombosis
Hb: Hemoglobin
HCT: Hematocrit
SD: Standard deviation
IQR: Interquartile range
ADL: Activities of Daily Living
SR: Sport and Recreation Function
FEV1: Forced expiratory volume in the first second
FVC: Forced vital capacity
WBC: White blood cell
PLT: Platelet
LY: Lymphocyte
ESR: Erythrocyte sedimentation rate
CRP: C-reactive protein
LDH: Lactate Dehydrogenase
ALT: Alanine Aminotransferase
AST: Aspartate Aminotransferase
CK: Creatine Kinase
mHHS: Modified Harris Hip Scores
APS: Antiphospholipid syndrome
SLE: Systemic lupus erythematosus
